# Dorothy Hodgkin lecture 2023: The enteroendocrine system—Sensors in your guts

**DOI:** 10.1111/dme.15212

**Published:** 2023-09-15

**Authors:** Frank Reimann

**Affiliations:** ^1^ Department of Clinical Biochemistry Institute of Metabolic Science & MRC Metabolic Diseases Unit, Addenbrooke's Hospital, University of Cambridge Cambridge UK

**Keywords:** diabetes, enteroendocrine, glucagon‐like peptide‐1, glucose‐dependent insulinotropic polypeptide, gut hormone, obesity, peptideYY

## Abstract

Glucagon‐like peptide‐1 (GLP‐1)‐based medication is now widely employed in the treatment of type 2 diabetes and obesity. Like other gut hormones, GLP‐1 is released from eneteroendocrine cells after a meal and in this review, based on the Dorothy Hodgkin lecture delivered during the annual meeting of Diabetes UK in 2023, I argue that there is sufficient spare capacity of GLP‐1 and other gut hormone expressing cells that could be recruited therapeutically. Years of research has revealed several receptors expressed in enteroendocrine cells that could be targeted to stimulate hormone release: although from this research it seems unlikely to find agents that selectively boost GLP‐1, release of a mixture of hormones might be the more desirable outcome anyway, given the recent promising results of new peptides combining GLP1‐receptor with other gut hormone receptor activation. Alternatively, the fact that GLP‐1 and peptideYY (PYY) expressing cells are found in greater density in the ileum might be exploited by increasing the delivery of chyme to the distal small intestine.

1

**What's new?**

- Glucagon‐like peptide‐1 receptor (GLP1R‐) based therapies are now established for the treatment of type2 diabetes and, at higher doses, for the treatment of obesity, with emerging drugs combining GLP1R activity with action at other gut hormone receptors.
- Research has identified a range of receptors expressed by enteroendocrine cells, the intestinal source of gut peptides including GLP‐1, which might be targeted pharmacologically to recruit endogenous hormone reserves.
- Shifting absorption of food to more distal intestinal areas might also result in a beneficial postprandial enteroendocrine plasma profiles.

## 2 INTRODUCTION

**FIGURE 1:**
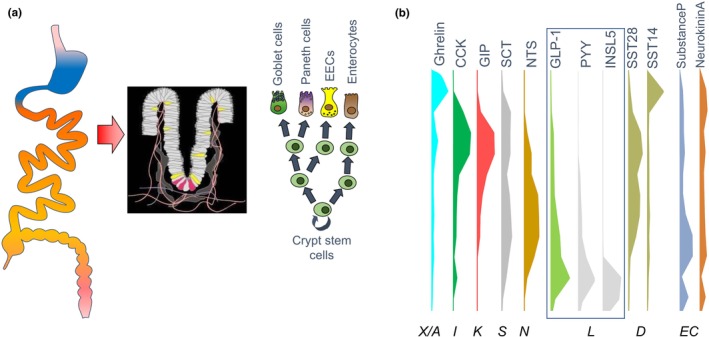
Enteroendocrine cells—rare cells found scattered in the intestinal epithelium expressing region specific hormones. (a) Enteroendocrine cells (EECs) arise from intestinal stem cells found at the bottom of the crypts, just like all other epithelial cells, however, only constitute ~1% of the epithelium. (b) Despite arising from pluripotent stem cells, EECs arising in different intestinal segments express different hormones, with ghrelin being dominant in the stomach, whereas for example GIP and CCK are found at highest amount in the proximal (duodenum/jejunum) small intestine. The figure is based on mass spectroscopic analysis of intestinal tissue samples taken along the gut longitudinal axis as reported in [53].

When Dorothy Crowfoot Hodgkin revealed the structure of porcine insulin in 1969, 35 years after first embarking on the project, she paved the way for the development modern insulins used today. While insulin is still the only option in patients lacking endogenous insulin production, the concept that the intestine secretes factors that boost insulin secretion after food ingestion, for which Jean Claude Barre is credited to have coined the term ‘incretin effect’ in 1932, is the foundation for more recently introduced therapeutic options for patients with residual pancreatic beta‐cell function. The incretin effect, the phenomenon that an equivalent plasma glucose rise triggers substantially more insulin release when the glucose was ingested orally than delivered intravenously, has been shown to depend on two gut hormones—glucagon‐like peptide‐1 (GLP‐1) and glucose‐dependent insulinotropic polypeptide (GIP)—which are released during the process of small intestinal glucose absorption, and potentiate glucose stimulated insulin secretion from pancreatic beta cells. Therapies based on GLP‐1 receptor (GLP1R) agonism are now major players in the treatment of type 2 diabetes and obesity. The gut, however, is the source of ~20 different hormones (see examples in Figure 1) that shape and report the progress of food digestion and nutrient absorption. The success of GLP‐1 based therapies has brought their cells of origin, the enteroendocrine cells (EECs), into the limelight of research—questions still not fully answered are whether we could recruit endogenous GLP‐1 pools and/or if we could modulate the release of other gut hormones therapeutically.

## 3 THE RISE OF GLP‐1‐BASED THERAPEUTICS

GLP‐1 was discovered in the late 1980s as an additional conserved sequence within the proglucagon molecule. It is produced from specialised enteroendocrine L‐cells as a mature bioactive peptide (either GLP‐1[7–36amide] or GLP‐1[7–37]), which is rapidly inactivated in the circulation by dipeptidyl peptidase 4 (DPP4) (see Drucker & Holst^1^ for a recent review). GLP‐1‐producing L‐cells are scattered throughout the epithelium of the small and large intestines, with the highest L‐cell densities in the distal ileum and colon. The other incretin, GIP, is produced from a proGIP precursor in enteroendocrine K‐cells, a different and only partially overlapping enteroendocrine cell type found scattered at high density in the upper small intestinal epithelium, and is similarly inactivated by DPP4.^1^

The idea of developing GLP‐1‐based therapies for type2 diabetes mellitus (T2DM) crystallised following the demonstration that overnight GLP‐1 infusion in people with T2DM raised insulin concentrations, suppressed glucagon release and normalised plasma glucose levels.^2^ A flurry of follow‐up studies highlighted the ability of supraphysiological GLP‐1 levels to stimulate first‐ and second‐phase insulin secretion, which in isolated islet studies was shown to be attributable to a stimulation of cAMP generation in beta cells downstream of the GLP1R. An important feature of this beta cell mechanism of action is that elevated cAMP only stimulates insulin release in the presence of an additional stimulus like glucose that causes membrane depolarisation. The critical consequence is that the stimulatory effect of GLP‐1 on insulin release switches off at low glucose levels, underlying the low incidence of hypoglycaemic side effects associated with the clinical use of GLP‐1‐based therapies.^3^ Also importantly, continuous intravenous GLP‐1 infusion over 6 weeks in people with T2DM^4^ resulted in prolonged lowering of plasma glucose levels, suggesting that long term targeting of the GLP1R would not be associated with loss of drug efficacy due to receptor downregulation.

In developing GLP‐1‐based therapeutic strategies, the most important consideration was the inactivating effect of DPP4,^5^ which limits the half‐life of bioactive endogenous GLP‐1 to only a few minutes. DPP4 inhibitors were developed that stabilise native GLP‐1 by preventing its inactivation, and synthetic peptides directly targeting GLP1R were selected on the basis of their DPP4 resistance and plasma stability. Now, 30 years later, we have highly efficacious long acting GLP1R agonist peptides such as semaglutide, with previously unmatched ability to lower plasma glucose and reduce body weight in the overweight type 2 diabetic population.^6^, ^7^ The other incretin, GIP, by contrast was somewhat neglected as a therapeutic option, as early research showed an inferior activity of this hormone compared to GLP‐1, even when infused at supraphysiological concentrations^8^; this is commonly considered to reflect loss of GIP‐sensitivity in patients with type 2 diabetes, possibly as a consequence of chronically elevated plasma glucose.^9^ Newer drugs such as tirzepatide, however, combine GLP1R agonism with GIPR agonism and have even more profound effects on body weight and blood glucose,^10^ and so‐called triple agonists combining GLP‐1, GIP and glucagon receptor activity are currently under development that show even greater promise with regard to weight loss.^11^, ^12^, ^13^

## 4 LESSONS FROM BARIATRIC SURGERY AND MOUSE MODELS

The success of GLP‐1‐ and GIP‐based therapies raises the question of whether similar outcomes could be achieved by targeting small molecules to the enteroendocrine L‐ and K‐cells, to increase release of endogenous stores of these hormones. A prerequisite for this approach is that intestinal reserves of GLP‐1 and GIP must be sufficiently high so that their recruitment could beneficially modify plasma glucose levels in the context of diabetes. An interesting case study in a patient having undergone Roux‐en‐Y gastric bypass surgery, who was suspected to have developed leakage from the gastric pouch/jejunal anastomosis and therefore had been fitted with a gastric catheter into the stomach remnant attached to the Roux limb, demonstrates the importance of the route of nutrient delivery for gut hormone release^14^—when given a liquid meal through the gastric catheter, enabling nutrient digestion and absorption within the Roux limb, this patient demonstrated elevated blood glucose levels 2 h after the meal, with plasma GLP‐1 and insulin excursions in the normal range, despite some weight loss at the time of the investigation. On the adjacent day, when the meal was ingested orally and thus rapidly delivered to the lower small intestine, where it mixed with the digestive juices delivered from the gallbladder and pancreas through the Roux limb, the same patient's glucose levels returned to baseline more rapidly which correlated with strongly elevated GLP‐1 and insulin responses. The strongly elevated GLP‐1 and PYY responses are not limited to this case. The majority of bariatric procedures, with the exception of gastric banding, are associated with elevated post‐prandial secretion of GLP‐1 and PYY. After profound weight loss in response to bariatric surgery or in lean gastric cancer patients with a RYGB‐like anatomy post‐gastrectomy, post‐prandial hypoglycaemia is an increasingly recognised complication. This appears to reflect large post‐prandial insulin responses in the presence of improved or normal insulin sensitivity and is reversed by GLP1R antagonism,^15^, ^16^, ^17^ indicating a physiological role for GLP‐1 in this context. Exendin‐9, under the name avexitide is currently under development for the treatment of post‐bariatric post‐prandial hypoglycaemia,^18^ and linkage of fatty acids to exendin‐9, similar to the modifications improving receptor agonists pharmacokinetics might be possible, with GLP‐1R antagonising antibodies presenting a potential alternative approach.^19^, ^20^ Questions about the importance of GLP‐1 and PYY for the metabolic benefits of bariatric surgery have, however, been raised in rodent models, as animals with knockout or pharmacological blockade of GLP1R or PYY receptors (NPY2R) still exhibited weight loss and improved intraperitoneal glucose tolerance after gastric bypass surgery.^21^, ^22^ PYY action through other NPY receptors in pancreatic islets has been linked to increases in beta cell mass^23^ and improved glucose homeostasis after bariatric surgery,^24^, ^25^ but it is difficult to establish the relative contribution of these mechanisms in the context of improved insulin sensitivity after weight loss. Nevertheless, the findings in human bariatric patients indicate not only that endogenous GLP‐1 and PYY reserves are sufficient to sustain high post‐prandial plasma concentrations even months and years post‐surgery and often after prolonged presurgical history of diabetes, but also that at least in the context of preserved beta cell function and insulin sensitivity, the elevated GLP‐1 levels can promote insulin secretion and lowering of plasma glucose. The lack of concordance with rodent models might reflect a greater contribution of other processes, including inflammation to the post‐operative weight loss in rodents.

Modern bariatric procedures are associated with complete nutrient absorption, although the digestive and absorptive processes are shifted to lower regions of the small intestine. Two major anatomical changes underlie this shift. First, most bariatric procedures interfere with the normal function of the stomach as a food reservoir with a regulated rate of gastric emptying, so rapid nutrient fluxes into the small intestine overcome the capacity of the upper gut to digest and absorb the incoming nutrient load. Second, a number of operations like RYGB involve the generation of a biliopancreatic Roux limb as a conduit for secretions from the pancreas and gallbladder, with the consequence that digestion of fats and proteins that rely on bile and pancreatic enzymes can only commence at sites distal to the anastomosis with the alimentary limb. Nevertheless, provided there is a sufficient length of common limb distal to the anastomosis, food absorption can be completed in the small intestine without development of malabsorption and losses into the colon.

From the perspective described above, it is rather surprising that the colon and rectum contain their own large reservoirs of GLP‐1‐ and PYY‐producing cells, even though these regions should never make contact with ingested nutrients. This raises several interesting questions, including: what are the physiological stimuli of colonic and rectal L‐cells? Would these large intestinal reserves of GLP‐1 and PYY, if recruited pharmacologically, be potentially capable of modulating metabolism and appetite? The answer to the first question may be that the colon contains L‐cell stimuli other than nutrients, including bile acids,^26^ short‐chain fatty acids^27^ and likely other stimulatory factors from the microbiota, which may determine basal levels of GLP‐1 and PYY secretion in the inter‐prandial state. To address the second question, we examined the physiological consequences of stimulating only the L‐cell population in the second half of the colon/rectum using a mouse model exploiting the distally‐restricted L‐cell expression of insulin‐like peptide‐5 (INSL5). Chemogenetic stimulation of Insl5‐expressing L‐cells using designer receptors activated by designer drugs (DREADD) resulted in acute GLP1R‐dependent lowering of blood glucose and NPY2R‐dependent suppression of food intake, validating that even GLP‐1 and PYY released from the distal gut can exert beneficial metabolic activity in vivo.^28^

## 5 INTESTINAL L‐CELLS AS A PHARMACOLOGICAL TARGET

**FIGURE 2:**
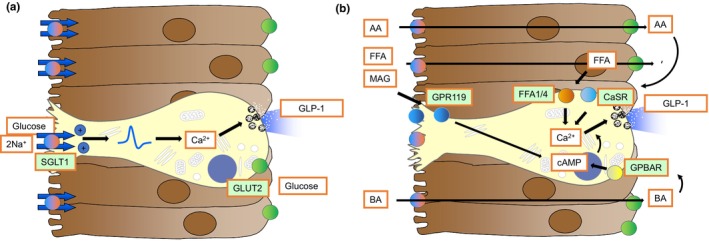
Stimulus secretion coupling in L‐cells. (a) Glucose‐sensing L‐cells employ the same apical glucose transporter as the surrounding enterocyte to absorb glucose from the intestinal lumen. The sodium co‐transport through SGLT1 (sodium‐coupled glucose transporter‐1) is sufficient to depolarise the L‐cell plasma membrane to activate voltage gated ion channels, culminating in the recruitment of voltage‐gated Ca^2+^‐channels and the resulting rise in cytosolic Ca^2+^‐concentrations triggering secretory vesicle release. (b) Other post‐prandially luminal compounds, such as bile acids, amino acids, fatty acids and mono‐acyl‐glycerides, despite electrogenic or electroneutral transporters being expressed in L‐cells, employ a different mechanism. Transporters expressed in the surrounding enterocytes mediate absorption and appearance of nutrients at the basolateral side, where they can activate G‐protein‐coupled receptors initiating elevation of cytosolic Ca^2+^ or cAMP, with the latter increasing Ca^2+^‐dependent vesicular release. Basolateral location for FFA1/4, CaSR and GPBAR has been indicated by experimental evidence, but apical and basolateral location for the MOG‐receptor GPR119 has been reported. FFA appearance at the basolateral side might involve chylomicron formation and secretion.^54^ AA, amino acids; BA, bile acids; CaSR, Ca‐sensing receptor, which also is sensitive to AA; FFA, free fatty acids; FFA1/4, free fatty acid receptors 1 and 4; GPBAR, G‐protein‐coupled bile acid receptor (also known as TGR5); GPR119, G‐protein‐coupled receptor 119, a MAG‐receptor; MAG, monoacyl‐glyceride; SGLT1, sodium‐dependent glucose transporter 1.

L‐cells, like the majority of EECs in the intestine, are open‐type endocrine cells with an apical face contacting the gut lumen and a basolateral region containing abundant secretory vesicles (Figure 2). Hormone release is triggered by regulated vesicular exocytosis, promoted by elevations in cytoplasmic Ca^2+^ and cAMP. Physiologically, GLP‐1 release is observed following ingestion of carbohydrates, fats and/or proteins, which must be digested to their component monosaccharides, fatty acids, monoacylglycerols, amino acids and di/tripeptides as an obligatory step to generate L‐cell stimuli. The molecular mechanisms underlying L‐cell stimulus detection have been uncovered using cell lines, transgenic mouse models and engineered intestinal organoids.^29^ Identifying which cells to study has been a critical step in the evolution of this research field, as at the resolution of light microscopy, live EECs can only be distinguished from their enterocyte neighbours by expression of a cell specific marker such as a fluorescent protein. Transgenic mice^30^ or genetically engineered human intestinal organoids^31^, ^32^ expressing fluorescent tags regulated by the proglucagon promoter have enabled the identification, purification and characterisation of L‐cells generated in native intestines or cultured ‘mini‐gut’ organoid model systems.

The application of RNA sequencing to purified EECs has revealed the richness of the sensory machinery expressed in these cell types. Unlike many other sensory cells in the body which respond to a single stimulus modality, EECs express a range of nutrient‐responsive sensory proteins including substrate transporters and G‐protein‐coupled receptors (GPCRs). Years of research spanning mouse and human EEC models have identified a variety of key stimulus‐receptor pairs involved in post‐prandial gut hormone secretion. GPCRs responsive to long chain fatty acids (free fatty acid receptors 1 and 4, FFA1/4 also known as GPR40 and GPR120, respectively), monoacylglycerols (GPR119), aromatic amino acids (calcium‐sensing receptor, CaSR) and bile acids (GPBAR1 also known as TGR5) are highly and differentially expressed by L‐cells, and their activation has been linked to elevation of Ca^2+^ (FFA1, CaSR) or cAMP (GPR119, GPBAR1) in primary L‐cells following ligand application in vitro, leading to the stimulation of GLP‐1 release.^33^ An anomaly is glucose, which relies on neither a GPCR nor the pancreatic islet K_ATP_ channel for its detection, but instead adopts the brush border sodium‐coupled glucose transporter SGLT1 to generate an L‐cell electrical signal proportional to the local rate of glucose absorption.^34^ So far, no major differences have been observed between mouse and human L‐cells with respect to stimulus responsiveness in vitro and in vivo, or sensory receptor expression profiles.

## 6 L‐CELL DISTINCTIVENESS AMONG THE WIDER CATEGORY OF EECs AND CONSIDERATIONS ON HOW TO TARGET THEM

L‐cells are categorised morphologically by their immunostaining for proglucagon (GLP‐1) and PYY, but are otherwise very similar in microscopic appearance and transcriptomic expression profile to EECs expressing other hormones such as GIP, cholecystokinin (CCK) and neurotensin. The subtleties separating one EEC population from another are apparent, however, when examining clustering patterns derived from single‐cell RNA sequencing data. Because the total EEC population makes up only ~1% of the intestinal epithelium, single cell RNA seq analysis without any prior cell enrichment step has previously covered too few EECs for cluster analysis. Small intestinal EECs purified from NeuroD1‐Cre mice (labelling all EECs)^35^, ^36^, ^37^ or neurogenin3‐labelled human organoids^32^—in this case neurgenin3 overexpression drove EEC differentiation—analysed by scRNAseq have, however, yielded thousands of cells for clustering. The patterns emerging from this approach are that K‐cells (GIP), D‐cells (Somatostatin) and enterochromaffin cells (5‐HT) form their own unique cell clusters, but that cells producing CCK, GLP‐1, PYY and neurotensin comprise a large overlapping cluster with frequent co‐expression of multiple hormones in a single cell. Even hormones mostly found in distinct clusters can, however, be co‐expressed in individual cells as exemplified by a small population found to express Tph1, the critical hormone for serotonin production, and proglucagon, giving rise to GLP‐1, in the duodenum.^38^

Transcriptomic analysis of cells labelled downstream of the Cck,^39^ Gip^40^ or Gcg^30^ promoters have repeatedly identified the same profiles of differentially‐expressed nutrient and bile acid sensitive GPCRs. From this perspective, it would be predicted that agonists (physiological or pharmacological) targeting FFA1, GPBAR1, CaSR or GPR119 would trigger a mixed hormonal response in vivo, including release of GLP‐1, PYY, CCK, GIP and neurotensin. It is quite difficult to disentangle from the literature whether all these hormones really rise in parallel in response to every nutritional challenge, since most studies have measured only one or a small number of hormones, and between‐study comparisons are complicated by differences in experimental design. In the post‐prandial situation, the temporal profiles of different gut hormones are determined at least in part by the predominant sites of hormone expression: since CCK and GIP are found in high amounts in the duodenum, they are stimulated rapidly after gastric emptying, whereas ingested foods need to penetrate further down the gut to reach the densest populations of cells producing GLP‐1 and PYY. A recent report measuring several gut and pancreatic derived hormones in parallel with frequent sampling before and after a liquid meal came to similar conclusions.^41^ Importantly, PYY and GLP‐1 trigger the so‐called ileal brake, slowing down delivery of chyme from the stomach to the small intestine, increasing the chance of nutrient absorption before overspill into the ileum, thereby limiting arrival of their own secretory stimuli during a meal.

From a therapeutic perspective, the transcriptomic and functional data suggest that small molecules targeting the GPCRs described above would not distinguish between cell types producing CCK, GIP, GLP‐1, PYY or NTS, except through pharmacokinetic properties differentially favouring drug access to a particular cell population such as the large vs small intestine. A mixed hormonal response might, however, be favourable, as recent research has hinted toward context‐dependent outcomes of individual hormone elevation, such that CCK, traditionally considered a hormone with short‐lived anorexic properties, promotes feeding of calorific sugar or sugar/fat meals.^42^, ^43^ While this could be hardwired into the neuronal afferent pathway of individual EECs, this seems fairly unlikely and other clues, like plasma nutrient context and other plasma hormone levels might influence the outcome of stimulating release of one hormone. In support of such complexity, GIP seems to counterbalance the nauseating effects of sudden rises in GLP‐1^44^ or PYY,^45^ at least when given at pharmacological doses, with signals converging at the area postrema, a part of the brain with a ‘leaky’ blood–brain barrier and importance in nausea perception modulation by GIP.^46^ As different EEC populations also express receptors for gut hormones predominantly arising from other EECs, one might even speculate on the existence of an epithelial integration shaping the hormonal signal triggered by different meals—future research will need to monitor hormones in parallel rather than concentrating on one or two to address these possibilities. A notable common feature of EEC‐receptors is their directional responsiveness. Studies in Ussing chambers or perfused intestinal preparations have arrived at similar conclusions—that agonists for FFA1,^47^ GPBAR1^26^ and CaSR^48^ on EECs are most effective when applied from the basolateral or vascular direction, requiring either local drug absorption through the epithelium adjacent to the EEC or access via the circulation. To limit systemic exposure—many of these GPCRs are also expressed in other tissues, stimulation of which might result in unwanted side effects—molecules with limited bioavailability due to first pass liver metabolism are likely the best option.

**FIGURE 3:**
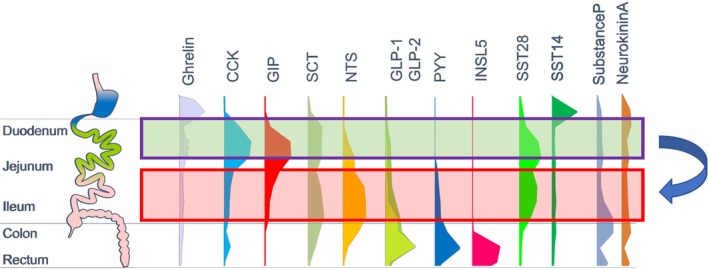
Shifting nutrient absorption to the lower small intestine—a therapeutic possibility. Post‐prandial nutrient digestion and absorption is usually limited to the proximal small intestine (duodenum/jejunum) (*green rectangle*). In fact, penetration of chyme to the more distal small intestine (ileum) triggers the ileal brake, limiting further delivery from the stomach, which is mediated at least in part by GLP‐1 and PYY. Shifting nutrient absorption to the more distal intestine (*red rectangle*) results in a different post‐prandial hormonal profile, with prolonged elevation of plasma GLP‐1/PYY levels, which contributes to the metabolic improvements seen after bariatric surgery and pharmacological inhibition of nutrient digestion (*see text for details*).

As outlined above, L‐cells are found in higher density in the more distal small intestine and their release of GLP‐1 and PYY release underlies the ileal brake. Bariatric surgery shifts nutrient absorption to more distal parts of the small intestine, which is associated with profoundly exaggerated post‐prandial GLP‐1 and PYY plasma levels, presumably attempting to pull the ileal brake. An alternative to the pharmacological targeting of L‐cell receptors might thus be simply shifting where nutrients are absorbed (Figure 3). In fact, this might well contribute to some medications already exploited for the treatment of diabetes, such as orlistat, which inhibits intestinal lipid digestion, and arcabose, miglitol and voglibose, which as alpha‐glucosidase inhibitors shift disaccharide digestion to the more distal intestine. All of these have at least in some studies been linked to elevated GLP‐1 levels, which might contribute to their therapeutic benefit, although complex interplay with gastric emptying and the degree to which enzymatic action is inhibited might result in augmented or reduced post‐prandial GLP‐1 responses, as has been discussed for orlistat.^49^, ^50^ Partial inhibition of glucose absorption with the mixed SGLT‐1/SGLT2 inhibitor sotagliflozin similarly shifts the site of glucose absorption; the slower glucose delivery to the blood due to the SGLT1 inhibition, the glucose transporter employed by absorptive enterocytes, itself is likely to limit post‐prandial glucose excursions, but this also triggers substantial GLP‐1 release at time points late after glucose ingestion.^51^, ^52^ In pilot data from our laboratory, we found that similar late GLP‐1 responses are also triggered in mice after oral administration of l‐ rather than d‐glucose, suggesting that delivery of non‐nutritive compounds to the more distal small intestine also can trigger late substantial GLP‐1 and PYY release (manuscript in preparation). Exploiting the yet to be identified molecular mechanisms might give rise to alternative treatment regimes, but of course, just like the already established lipase and glucosidase inhibitors, might have unwanted intestinal side effects, tolerable by some, but not other patients.

## 7 CONCLUSION

In this review, based on the content of my Dorothy Hodgkin prize lecture, I have tried to present the evidence that a ‘reserve pool’ of intestinal hormones, especially GLP‐1 and PYY, is recruitable in patients to treat post‐prandial glucose excursions and that these likely contribute to weight loss seen after bariatric surgery. Research in Fiona Gribble's and my laboratory has identified many receptors on EECs and characterised the molecular mechanisms linking their activation to hormone release. While these present obvious yet to be exploited pharmacological targets for the treatment of diabetes and obesity, other interventions that shift nutrient delivery and absorption to the distal small intestine also should be pursued.

## CONFLICT OF INTEREST STATEMENT

I have no conflict of interests.

## Data Availability

Data sharing is not applicable to this article as no new data were created or analyzed in this study.
